# A management perspective on resilience in healthcare: a framework and avenues for future research

**DOI:** 10.1186/s12913-023-09701-3

**Published:** 2023-07-19

**Authors:** L. Agostini, R. Onofrio, C. Piccolo, A. Stefanini

**Affiliations:** 1 Department of Management and Engineering, University of Padova, Stradella San Nicola 3, Padua, Italy; 2 Department of Management, Economics and Industrial Engineering, Politecnico Di Milano, Piazza Leonardo da Vinci, 32 Milano, Italy; 3 Department of Industrial Engineering, University of Naples Federico II, C.So Umberto I, 40, Naples, Italy; 4 Department of Energy, Systems, Territory and Construction Engineering, University of Pisa, Lungarno Antonio Pacinotti, 43, Pisa, Italy

**Keywords:** Resilience, Healthcare, Literature review, Flexibility, Agility

## Abstract

Recent major health shocks, such as the 2014–16 Ebola, the Zika outbreak, and, last but not least, the COVID-19 pandemic, have strongly contributed to drawing attention to the issue of resilience in the healthcare domain. Nevertheless, the scientific literature appears fragmented, creating difficulties in developing incremental research in this relevant managerial field.

To fill this gap, this systematic literature review aims to provide a clear state of the art of the literature dealing with resilience in healthcare. Specifically, from the analysis of the theoretical articles and reviews, the key dimensions of resilience are identified, and a novel classification framework is proposed. The classification framework is then used to systematize extant empirical contributions. Two main dimensions of resilience are identified: the approach to resilience (reactive vs. proactive) and the type of crisis to deal with (acute shocks vs. chronic stressors). Four main streams of research are thus identified: (i) proactive approaches to acute shocks; (ii) proactive approaches to chronic stressors; (iii) reactive approaches to acute shocks; and (iv) reactive approaches to chronic stressors. These are scrutinised considering three additional dimensions: the level of analysis, the resources to nurture resilience, and the country context. The classification framework and the associated mapping contribute to systematising the fragmented literature on resilience in healthcare, providing a clear picture of the state of the art in this field and drawing a research agenda that opens interesting paths for future research.

**Supplementary Information:**

The online version contains supplementary material available at 10.1186/s12913-023-09701-3.

## Introduction

The concept of resilience is assuming an increasingly central role in the debate about healthcare systems overall, considering recent major health shocks, such as the 2014–16 Ebola outbreak, the Zika outbreak, and, last but not least, the COVID-19 pandemic that has recently brought an outburst of publications on this topic. However, these emergencies have only raised a problem that tends to be systemic in the healthcare sector [1–3], as unexpected changes and uncertain conditions, in terms of both disruptions and routine stressors, are progressively more frequent. Within this context, the lack of flexibility of health systems implies a poor ability to adapt to conditions other than standard ones and increased vulnerability, leading to a decline in performance. Therefore, resilience has been gaining momentum as one of the most effective answers to this situation [4].

The concept of resilience has been applied across multiple disciplinary fields, such as engineering, where resilience is intended as the capability of a system to build adaptive capacities when disturbances occur and then “bounce back” to the previous equilibrium, which is mainly used in the safety science area [5, 6]; ecology, which focuses on how biological systems and communities cope with uncertainties and maintain stability [7, 8]; psychology, which concentrates on the ability of individuals and communities to develop and learn after external pressures and trauma; and the organisational management, which centres on how companies can act in response to rapidly changing business environment [9–12]. In this study, which has a managerial perspective, we adopt the definition of resilience “as the ability that individuals, communities, organisational units or larger systems have to return to some ‘normal’ condition or state of functioning after a disruptive event; to cope with pressure and problems by being flexible without compromising system performance; or to adapt to a new normal state, where system functioning is reorganised or enhanced in some way in response to the disruption they face” ([12], p. 3).

The lack of clarity on organising and dealing with this topic has led to fragmented scientific literature, thus creating difficulties in developing and assimilating incremental research in this relevant field, which pushes reviewing the literature to systematise the research in this area.

Nevertheless, extant management literature reviews on resilience in healthcare have several limitations. First, they do not offer any comprehensive classification tool to systematise the fragmented literature, thus being able to identify future research questions and avenues to foster the improvement, transparency, and quality of future research about resilience in healthcare. Second, most available reviews of the literature tend to be scoping reviews, rather than systematic, and generally have a narrow scope—for example, answers to shocks, everyday resilience, Ebola outbreaks in West Africa, and developing countries [13]. Third, the analyses frequently consider a small number of articles [14–16], thus leading to a shortage of comprehensive frameworks guiding the development of literature in the field, or limited to a commentary or perspective on the topic [17, 18]. Lastly, the recent sharp increase in publications in this domain, overall in light of the COVID-19 emergency, is likely to have contributed significantly to the body of knowledge, but it has not yet been covered by past reviews.

To fill this gap, the aim of this research is twofold: to design a multi-dimensional classification framework for resilience in healthcare, trying to organise the current literature on the topic; and to provide a clear, overarching, and updated picture of the current state of the art about resilience in healthcare and allow the identification of promising future research avenues. On this basis, we address two specific research questions:

- RQ_1_: What are the key dimensions that can be used to systematise the literature on resilience in healthcare?
- RQ_2_: What are the most significant evidence on resilience in healthcare, and what are the most promising avenues for future research?

This study aims to answer these research questions by applying a systematic literature review of 178 articles.

Building on the existing conceptual framework of resilience [2, 13, 19], we identify the key dimensions to classify the existing empirical literature, which allows us to design the classification framework and the associated mapping of empirical articles. Their critical analysis allows an overview of extant literature, summarises the ongoing trends on resilience in healthcare, highlights the existing gaps, and recommends promising avenues for future research, thus providing a knowledge base for further scientific development in this relevant research area.

The remainder of the article is organised as follows. Methodology section provides a description of the methodology used to conduct the systematic literature review. Descriptive Analytics of the Sample section illustrates the descriptive analysis of sampled articles. Content Analysis: Theoretical and Review Articles section enters into the details of the theoretical and literature review articles from which the framework was obtained, and Content Analysis: A Cross-Dimensional Analysis of Empirical Articles section presents the analysis of empirical articles based on such a framework. Finally, Discussion and Avenues for Future Research section illustrates promising directions for further research, and Conclusion section provides some concluding remarks.

## Methodology

To reach our purpose, we followed a systematic and transparent method based on [20], which is designed to guide a literature review. As a first step, we aimed to identify the set of articles to be analysed. This implies beginning with a broad dataset to be progressively reduced in later steps. The starting database was defined by combining the keyword health*, searched in the title, with the keywords resilien* or flexib* or adapt* or readiness* or agil*, searched in the topic, in the scientific database ISI Web of Science (WoS) Core Collection in June 2022. We opted to complement the keyword resilien* with other terms to cope with the fact that different constructs are sometimes used with similar meanings, which is quite frequent in different streams of literature when they develop very rapidly [21], despite erroneous. Accordingly, we retained articles using different constructs that, however, were used with the same conceptual meaning as resilience.

This search produced 29,306 results. After refining the query by WoS category capturing the management domain (i.e. operations research management science, economics, management, business, healthcare sciences & services, and health policy and services), by document type (i.e. article, review, early access, or editorial material), and language (i.e. English), we ended up with 1066 articles.

As a second step, we read the abstracts and established specific exclusion criteria to leave non-pertinent articles out of the analysis. In particular, when the concept of resilience or the related ones were not considered at all or not with reference to a health organisation or service, the article was excluded from the sample (e.g. resilience of the patient, health intended as improving air quality or reducing soil consumption). When collateral subjects (e.g. lean principles, complex adaptive systems) were the main topic of the article, we retained it only if there was a relevant mention of resilience. We also maintained the same criteria for the third step, which consisted of full article reading of those contributions requiring further examination because the abstract was not sufficiently informative. After cross-checking the exclusion of articles in the final pool and reviewing the references of relevant articles (i.e. backward snowballing), we obtained 178 articles that are the object of investigation of this analysis.

As a final step, we analysed the content of those articles carefully and mapped in a spreadsheet the main fields of interest, namely the purpose of the research, the methodology employed, the context of analysis, the research questions/hypotheses, the theoretical background, the main constructs and associated definitions, the key findings, and future research. As the examination of theoretical articles allowed the identification of five key dimensions of resilience in healthcare, as presented in Content Analysis: Theoretical and Review Articles section, empirical articles were also mapped according those dimensions.

**Fig. 1:**
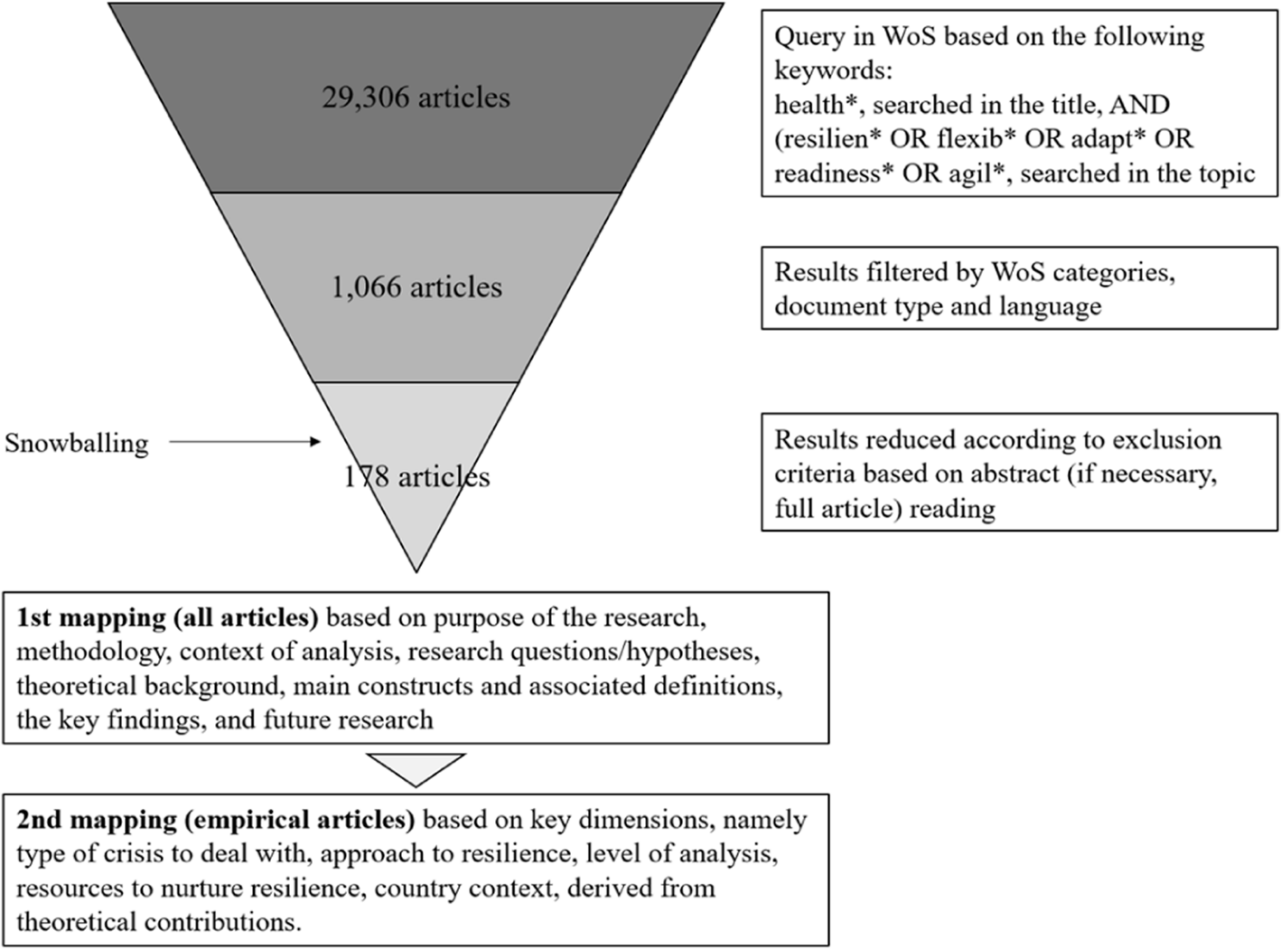
Methodological process

 Figure 1 summarises the overall methodological process.

## Descriptive analytics of the sample

**Fig. 2:**
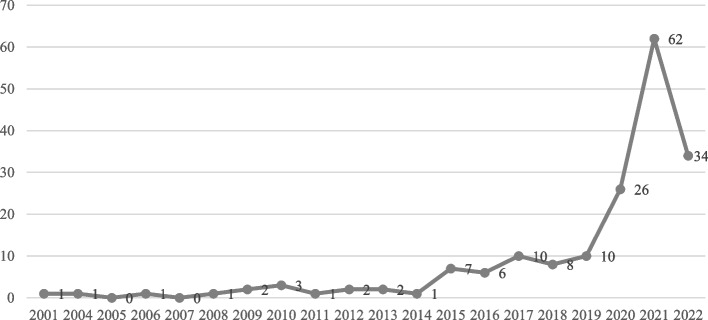
Annual scientific production

The sample’s 178 articles were published from 2001 to 2022. As shown in Fig. 2, the most significant increase occurred in the last 5 years (about 80% of documents sourced), particularly in the years 2020–2021. The strong rise in 2020 is only partially related to COVID-19, as 6 articles out of 26 focus on the pandemic (e.g. [4]), while the peak of the 2021 (number of publications is more than double compared to 2020) is almost entirely due to COVID-19 and it is expected to produce a further surge in research in the next few years.

The variety of outlets that publish articles dealing with resilience in healthcare is wide (i.e. 94 journals). Table 1 reports scientific journals with more than two articles in the sample. Among the 15 journals listed in the table, 9 belong to the first quartile of the SJR classification and 4 to the second quartile. These journals belong to different research areas, including “engineering, industrial operations research & management science, development studies, economics, health policy & services”, according to WoS categories. The two leading journals (i.e. “Health Policy and Planning” and “BMC Health Services Research”) fall within the category of “health care sciences & services”.

**Table 1:** Number of articles per journal

Scientific Journal	N. of Articles	SJR
Health Policy And Planning	16	Q1
BMC Health Services Research	10	Q1
Safety Science	7	Q1
Health Research Policy And Systems	7	Q1
International Journal Of Health Policy And Management	5	Q1
Journal Of Health Management	5	N/A
International Journal For Quality In Health Care	4	Q2
Bmj Quality & Safety	3	Q1
World Development	3	Q1
Disaster Prevention And Management: An International Journal	3	N/A
Australian Health Review	2	Q2
Journal Of Evaluation In Clinical Practice	2	Q2
Medical Teacher	2	Q1

**Fig. 3:**
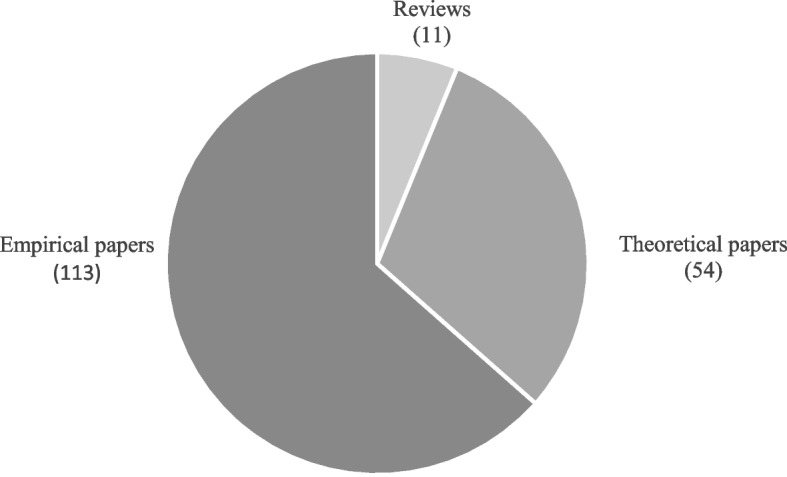
Classification of papers according to the adopted methodological approach

In Fig. 3, the articles are grouped according to the adopted methodological approach, that is, (1) theoretical articles, (2) literature reviews, and (3) empirical articles. The contributions in the first group theoretically discuss the concept of resilience in healthcare [22]; those in the second group review past literature on the topic, providing the state of the art from different perspectives and at various stages of research development [11]; finally, articles in the third group base their findings on primary or secondary (i.e. collected in other studies) empirical observations [23].

The analysis of *theoretical* and *review articles* sheds light on and clarifies the concepts, theories, and frameworks debated in the literature and allows for detecting the analysis dimensions for appropriately classifying the empirical research. The evaluation of *empirical articles *through the defined analysis dimensions allows understanding of the current status of resilience in healthcare research and identifying the most neglected areas. The results of the content analysis are reported in the next sections; specifically, Content Analysis: Theoretical and Review Articles section debates the theoretical papers and the reviews and illustrates the key dimensions of resilience that emerged from the literature, while Content Analysis: A Cross-Dimensional Analysis of Empirical Articles section discusses the empirical articles and their mapping according to the proposed classification framework.

## Content analysis: Theoretical and review articles

This section reports the main findings arising from the analysis of the 65 *theoretical* and *review articles.* A thorough examination of the articles reveals one of the primary concerns as the conceptualisation and definition of the resilience construct for both theoretical and review articles [14, 15, 24, 25]. Despite numerous studies on the subject, there seems to be no consensus on a shared description of resilience in the healthcare sector.

A notable spike of theoretical articles emerged in the first months of the Covid-19 outbreak, when researchers provided viewpoints and commentaries on how the crisis was or should have been addressed [26–29]. Most frequently, these articles stress lessons learned (or that should have been learned) from the pandemic, dealing with one or some factors that seem particularly relevant to build resilience, such as leadership [18], coordination and collaboration at the system level [30], and well-being of the healthcare workforce [31]. Some other articles, on the contrary, seem to use the pandemic to draw attention to problems of healthcare systems that were relevant even before, such as the constrained and limited resources and the psychological resilience of staff [3, 32].

Finally, some articles, both literature review and theoretical articles, propose frameworks in an effort to conceptualise resilience and pinpoint its key aspects. Specifically, three main frameworks [2, 13, 19] seem to emerge.

Barasa et al. [2] presented a framework that categorises the resources that may nurture resilience in healthcare systems, with a first division in hardware and software. With hardware, the authors intend the main physical ‘building blocks’ of healthcare systems, such as human resources, finances, and infrastructure. Software includes *tangible software*—such as organisational systems, managerial procedures, and management knowledge and skills—and *intangible software*—such as relationships, values, norms, and similar social factors. Barasa et al. [2] also made a distinction based on the type of crisis being encountered, although they did not introduce this dimension into the framework.

Blanchet et al. [19] proposed a classification of the possible strategies (i.e. absorptive, adaptive, and transformative) and capabilities of healthcare systems to address the resilience issue, starting from previous frameworks from ecological science. The strategies are classified based on the intensity of change, from reactive to structural changes, and based on the type of crisis, from stress to shock. Thus, the authors acknowledged that beyond mere reactive, bouncing-back strategies to address isolated issues, the healthcare system has witnessed the development of more sophisticated strategies that structurally/proactively adapt them to potential upcoming changes.

Biddle et al. [13] slightly re-adapt the framework of Blanchet et al. [19] and exploit it for classifying the 40 empirical research studies they selected for the review. To this purpose, the authors even introduced the country context in their review, recognising its relevance. Biddle et al. [2] also analysed the frameworks already present in the literature, observing that, while there are some application frameworks useful for calculating resilience indexes [33], the need for a comprehensive assessment framework that organises the research addressing healthcare resilience strongly emerges. Although not integrating this dimension into a framework, Biddle et al. [13] noticed the importance of the organisational level when looking at the studies in this area.

By analysing these frameworks and the classifications adopted in the literature reviews and theoretical papers, it was possible to identify the key dimensions of analysis that can be used to classify research in the field of healthcare resilience.

### The dimensions of resilience – a classification framework

The dimensions that emerged as relevant from the existing literature are listed below.

- Type of crisis to deal with
- Approach to resilience
- Level of analysis
- Country context
- Resources to nurture resilience

#### Type of crisis to deal with

The first dimension of investigation refers to the *type of crisis to deal with*. As outlined by Barasa et al. [2], despite resilience being traditionally intended as the capacity of a system to respond to shocks, it may also refer to the response to chronic stressors and/or everyday challenges. Accordingly, we distinguish articles dealing with ***acute shocks***—that is, sudden and sharp events disrupting the normal functioning of the systems (e.g. infectious diseases, natural disasters)—from those dealing with ***chronic stressors***, which are less acute in terms of intensity but may occur with a higher frequency (i.e. operational failures) and/or deploy their effect in the long run (e.g. demographic and/or climate changes).

#### The approach to resilience

The approach to resilience can be proactive or reactive*,* with proactive referring to preparedness and reactive referring to recovery from turbulence [34]. Accordingly, we classify articles by distinguishing between the following:

- ***proactive approaches***, aimed at reducing the likelihood of occurrence of adverse events and/or at anticipating future threats before they are actually experienced.
- ***reactive approaches***, aimed at reducing the consequences of the adverse events once they have already occurred.

#### Level of analysis

Another critical dimension is represented by the organisational level considered in the investigation [13]. Indeed, the response to a crisis can be analysed from a *macroscopic* point of view, by focusing on the capacity built by whole systems (i.e. countries, communities), or from a *microscopic* point of view, by focusing on the capacity of single organisations (e.g. hospitals, health units) or even single teams or individuals to respond to adverse events.

By taking inspiration from ecological science, we refer to a multilevel perspective on organisational learning [35]. In this sense, the response of a system to a crisis has to be viewed as the result of the interaction of multiple factors at different levels:

- M1: Micro (individuals)
- M2: Meso-small (interpersonal/teams)
- M3: Meso-large (organisations)
- M4: Macro-small (groups of organisations, communities, networks)
- M5: Macro-large (country or groups of countries)

#### Country context

The fourth dimension concerns the country context in which resilience is investigated. Such a perspective helps in understanding whether there are threats affecting health systems at a global level (e.g. pandemics) or a local level (e.g. natural disaster, local outbreak), but also in identifying context-specific barriers or opportunities in the implementation of resilience approaches [13]. Since it is relevant, although not implemented in their frameworks, some reviews and theoretical articles consider this aspect in the classification of literature [13, 36]. Accordingly, along this dimension, we distinguish between *developed* and *developing countries*.^1^

#### Resources to nurture resilience

The fifth dimension of classification concerns the prevalent factors/resources considered enablers of resilience. As previously described, the framework proposed by Barasa et al. [2] classifies resources to nurture resilience into hardware and software, with the latter further divided into tangible and intangible. Starting from such a classification, we introduce more refined dimensions as follows:

- ***Hardware resources***
- ***Organisation and management resources***, further distinguished into: *(i) management tools and practices, (ii) organisational capabilities, and (iii) leadership and governance;*
- ***Social factors***

Hardware resources are captured by empirical articles exploring the role played by physical resources in enabling organisational resilience, such as the health workforce, and the availability of equipment, materials, and infrastructure. In the second category, the focus shifts to the role played by organisational and management resources. Here, the first sub-category considers papers focusing on the development of appropriate *management tools and practices* to effectively manage information within an organisation and/or to support decisions, with the final aim of improving system response or their preparedness to crisis. In the second sub-category, studies have focused on *organisational dynamic capabilities*—for example, flexibility and agility [37]—as enablers or facilitators of resilience as owned by the organisation as a whole. The third sub-category focuses on the role of leadership and governance of systems during crisis. Finally, in the last category, *social factors*, articles explore the role of social norms, behavioural aspects, and social interactions in fostering resilience.

**Fig. 4:**
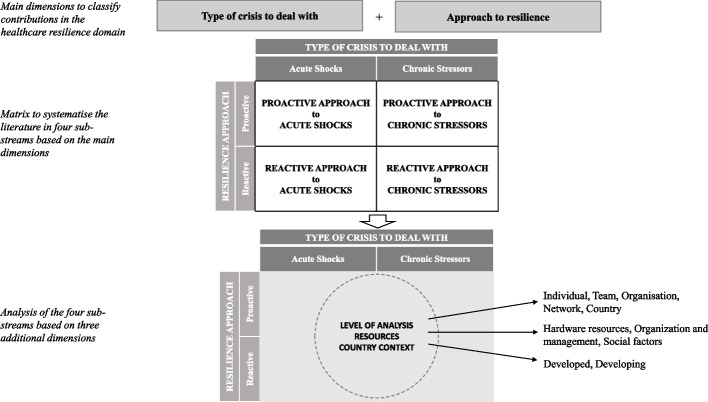
The classification framework

In our classification framework, we organised these dimensions according to a hierarchical structure. Specifically, the *type of crisis* and the adopted *resilience approach* were selected as the main dimensions of analysis and crossed to identify the four sub-streams of research shown in Fig. 4. Then, within each sub-stream, the other complementary dimensions, namely the *level of analysis*, the *country context*, and the *resources used to nurture resilience*, were analysed as contextual factors. The Additional file 1: Appendix presents a more detailed mapping.

## Content analysis: a cross-dimensional analysis of empirical articles

The classification framework introduced in the previous section represents a useful lens for analysing empirical articles dealing with resilience in healthcare and supports our systematisation of literature in this field. Subsequent sections present the empirical studies included in the four sub-streams identified above.

### Reactive approach to acute shocks

Studies in this group increased following the recent COVID-19 pandemic, with the research mainly set at the macroscopic level (M4 and M5). The authors proposed a set of factors associated with organisational and managerial aspects. More specifically, having an action plan that assures business continuity is a useful management tool that maximises its effectiveness if it goes beyond organisational boundaries, thus facilitating the coordination of different entities interested in a disaster [38]. This is strictly related to network capability, which is also considered a major requirement in developing countries [39]. Finding studies in developing countries is not surprising in this context, where an overarching response to disasters is common, which emphasises the need to make different actors, both public and private, interact in favour of the whole community [36, 40]. This also entails a social implication, which may be related to community engagement, involvement, and empowerment, along with a shared response perspective [41, 42].

This also holds true in developed countries, where a sense of safety, calming, efficacy, connectedness, and hope, thus embracing more a psychological sphere, has been proven to foster community resilience [8]. Following the COVID-19 pandemic, studies carried out in developed countries have increased sharply, stressing the low level of preparedness for such acute shocks, against the expectancy, of these countries. For this reason, authors have put forward some resources that could help further nurture resilience, such as strong but also participative governance [43, 44], increased training of capabilities [45], the use of data to make quick decisions on resource allocation [44, 46], and additive manufacturing to speed up the flexible manufacturing of products if needed [47]. This last evidence regarding digital technologies further supports the necessity of information systems, along with an information exchange community perspective that crosses boundaries to respond to a crisis effectively and efficiently, which was put forward before the COVID-19 outbreak [48]. Investments—both in managerial tools, in terms of master data management plans and data-sharing agreements, and in social factors, in terms of culture—are needed to sustain the infrastructure in developed countries [48]. In this stream, a study aimed at identifying mechanisms of adaptation to the COVID-19 crisis included staff taking on larger workloads, for example, using the existing service frameworks in new ways, shifting their services remotely and/or substantively, and utilising the trust they had built with communities and individuals over time [49].

On the side of developing countries, the authors still stressed the need for investments but more directly addressed the managerial domain and social capital to promote a sense of community, commitment, and professionalism [50].

At the organisational level (M3), it is also relevant to highlight that healthcare providers seem to show a low level of preparedness, in terms of knowledge and skill competencies, in being an emergency-resilient hospital, defined as an organisation that can resist, absorb, and respond to disasters as COVID-19 and simultaneously maintains its basic functions [51, 52]. For this purpose, recent research seems to point to both resources, in terms of workforce and funds [53], and organisational and managerial practices [22, 54, 55], which points to the need for a bundle of different resources interplaying for the same purpose. This evidence, combined with the scarcity of literature, confirms the need to carry out further research with the purpose of providing concrete indications and guidelines for how organisations can react to shocks. Lastly, at the micro level (M1), Raven et al. [56] stressed the social factors associated with workers’ ability to respond to shocks, with a particular focus on motivation, shared learning, and trusting relationships with the surrounding environment. At this level, the matter of capabilities, in particular adaptive, absorptive, and transformative [22, 57], remains at the core of personal resilience, together with a learning orientation and a stimulating working environment [58].

### Proactive approach to acute shocks

This second cluster of articles analyses proactive approaches for responding to shocks that, to varying degrees, can disrupt healthcare systems. As partially expected, this cluster has fewer articles than the previous one, since it appears more “natural” to respond to a shock by following a reactive approach. On the contrary, several authors have shown that it is better to develop a proactive approach able to anticipate, at least in part, the shock and its consequences [59–61].

At the organisational level, the selected articles show that the macro-large level (M5), followed by the macro-small level (M4), has attracted the most attention. For example, Connolly [62] investigated the policy-making challenges at the country level faced by the UK government in dealing with pandemic influences. The results underline how more proactive approaches may be beneficial in dealing with epidemic outbreaks. Unfortunately, the COVID-19 pandemic showed how unprepared health systems, both in developed and developing countries, were to proactively face an epidemic shock [61]. Landeg et al. [60] obtained similar results at the sub-national level assessing the healthcare system impacts associated with the December 2013 flooding in the Boston area of the US. Their work showed that healthcare systems appear to have a limited capacity to respond to weather-related impacts, particularly regarding reactive approaches, whereas better results may be obtained by adopting proactive action plans able to face unexpected situations.

The attention at the higher level of the organisational scale (M5 and M4) might be related to the fact that organisations, due to a naturally reduced vision, are more accustomed to reacting to the events while they are experiencing them, while communities and nations, having a higher-level vision and control, must proactively prepare healthcare systems for possible sudden shock. Nevertheless, few articles can also be identified at the micro level (M1), especially promoting proactive individual approaches at the psychological level for managing staff stress during health crises [63], a particularly prominent problem during the COVID-19 outbreak [64].

In the country context, we observe a strong prevalence of developed countries in this cluster, such as Australia [65], the UK [62, 66], the US [60], and Canada [63]. This result may depend on the fact that, in general, countries with fewer resources are focused on managing the current situation rather than on planning and carrying out proactive plans to cope with any potential shock.

Concerning the resources to nurture resilience, the literature focuses more on the factors associated with organisational and managerial aspects. Researchers are mostly interested in proposing managerial models that can proactively manage the shocks potentially affecting the healthcare system. These models propose strategies for managing all, or the majority of, the resources in the system, for example, human, equipment, and financial resources [65, 66]. Another topic of great interest is the investigation of governance strategies to proactively manage potential shocks. For example, Zarychta [67] assessed how a decentralisation management strategy may affect healthcare resilience in Honduras. Lastly, to a lesser extent, some articles focus instead on the exploitation of one or a few specific resources to cope with shocks in a proactive way.

### Reactive approach to chronic stressors

As expected, few articles belong to this group, since it appears harder and “less natural” to face chronic stressors, and their potential effect in the long run, with reactive strategies. In terms of the organisational level, the articles in this group mainly set their research at the micro (M1), meso-large (M3), and macro-large (M5) levels. In particular, at the organisational level (M3), two studies investigated specific resources to nurture resilience. The first one [68] considers two types of flexibility used to coordinate two important resources (beds and nursing staff) and satisfy stochastic demand at minimum cost. In this article, the authors explored the benefits and trade-offs of employing different types of flexibility while coordinating bed spaces and nursing staff. The second one [69] instead aims at identifying the key determinants of the healthcare system’s adaptive capacity to reactively respond to the outcomes—as rapid development of new technologies, sophisticated devices, and related applications—of the fourth industrial revolution in healthcare systems. The authors underlined that the most significant resources to leverage for this issue are human capital, financial resources, and legal regulations.

A similar flexibility can be seen at the macro-large level (M5). In this direction, Rodríguez-Álvarez et al. [70] focused on the flexible response of decentralised health services to demand uncertainty. At the micro level (M1), the focus is on individual capabilities. In particular, Desombre et al. [71] examined the increase in functional flexibility by employees through greater job variety and related training in three healthcare settings. The findings show that functional flexibility may improve service quality, despite some resistance from employees. At the level of the countries involved, we observe a strong prevalence of developed countries in this cluster, including the US [68], the UK [71], Spain [70], and Poland [71].

### Proactive approach to chronic stressors

Most of the contributions in this cluster focus on single health organisations (M3) and investigate the possibility of exploiting internal resources and capabilities to predict risk and prepare to proactively adapt to any potential change. In this context, many authors have developed management tools to support proactive decision making by employing data management, data visualisation, optimisation, and simulation techniques [72, 73]. For example, Ross et al. [74] proposed a model for holistic and real-time process monitoring within an emergency department. The proposed approach supports staff and organisations in anticipating and responding to variations in demand and pressure factors. Restrepo et al. [75] proposed a stochastic optimisation approach that integrates staffing and scheduling decisions in the context of home healthcare. This tool allows for making more robust decisions to accommodate changes in demand and to support the flexible planning of resources.

A second cluster of the articles analyses the internal sources of vulnerabilities to propose innovative safety improvement tools to prevent harm to patients. Traditionally, the focus for patient safety is to study incident reports and adverse events, but the new era of safety investigations calls for proactive approaches and the analysis of everyday clinical work [76–78]. For instance, Svensson and Bergström [79] introduced a new approach to system monitoring as a way to strengthen patient safety. The model uses a time-lapse visualisation of everyday ‘normal’ clinical work as a method to understand patterns of resilience and how risk could emerge in a psychiatric clinic.

Besides the management tools, these capabilities are also recognised as crucial to coping with chronic stressors. In this sense, Jack and Powers [80] focused on how healthcare organisations develop and leverage their resources to achieve volume flexible responses to demand variability. Rubbio et al. [81] analysed resilience mechanisms and capabilities in healthcare settings and attempted to understand how digital technologies may impact healthcare resilience. In this cluster, less attention has been devoted to the macroscopic analysis of healthcare systems (M4–M5).

Indeed, only a few studies have analysed proactive approaches undertaken at the country or community level. An example is represented by the evaluation of service readiness and ascertaining supply side barriers inhibiting service provisioning in rural, remote, and fragile districts [82]. Another example is represented by contributions focusing on the health risks associated with climate change. Climate change adaptation is arising as a new form of risk management and is posing new challenges for health systems, which are called to evaluate the impacts on the population’s health and to prepare for the potential effects on health service resources, the workforce, and infrastructure. There is a flourishing stream of new literature on coping with this emerging long-term chronic stressor. For example, Marcus and Hanna [83] conducted a cross-country analysis to assess national progress on climate change adaptation for public health and to identify the main barriers to the development of national adaptive capacity. The results show that the largest barriers to progress in this domain are poor government coordination, lack of political will, and inadequate adaptation finances. In this direction, Aracena et al. [84] called for new decentralised (regional) adaptation plans able to address the health impacts of climate change and fill in the policy vacuum that is currently present in developing countries like Peru.

Finally, the microscopic level (M1) also tends to be neglected. Among the contributions analysing the resilience of single individuals, Janes et al. [76] investigated the effects of training interventions designed to proactively prepare staff for coping with errors with an empirical case study.

## Discussion and avenues for future research

The content analysis of articles demonstrates that the literature has approached resilience to shocks (also called *shock resilience*) differently from resilience to chronic stressors (also called *everyday resilience*). First, a reactive approach is used more frequently to analyse how to deal with acute shocks than chronic stressors, for which researchers have already started investigating how to anticipate and, thus, be prepared in advance to face such events. It is not surprising to consider the nature of these two different types of events; indeed, acute shocks are more unexpected and more rare than chronic stressors. Therefore, practitioners, organisations, and countries are less prepared to face them and tend to adopt a reactive approach when they happen. The literature shows that several factors have proven effective in responding to acute shocks. In terms of resources, having funds available seems fundamental to facing unexpected events, as well as technological infrastructure and tools for telemedicine to replace in-person activities with remote ones. Following the same line, in terms of capabilities, rapid training is necessary to give employees the know-how to support this shift to remote activities, guided by strong leadership with effective communication abilities, on the one hand, and supported by rewarding and emotional support for employees, on the other, more social-oriented, hand.

However, the proactive side of resilience has progressively started to gain momentum in healthcare [85–87] and authors [25, 59] continue to stress the importance of anticipating changes. Overall, authors in the domain of chronic stressors have stressed the importance, on the one hand, to make use of management tools for optimisation or prediction to be ready to adapt conditions to changes in demand and, on the other hand, to have flexible resources to face demand variability. Accordingly, more research is needed in this area, and we encourage researchers to investigate how actors at different levels can be prepared to face such acute shocks and be resilient in the healthcare domain.

Second, the level of analysis of studies dealing with resilience to face acute shocks and chronic stressors is different, in the sense that shock resilience is addressed from a macro-large-level perspective (M5), that is, at the level of regional or national health systems. Instead, everyday resilience is studied mainly from the perspective of single organisations (M3). In line with this aspect, we find a prevalence of governance-related resources, beyond some general management tools (e.g. organisational systems), to nurture resilience when the level of analysis is the community or the whole country, and specific management tool-related resources (e.g. simulation or data visualisation tools) when the analysis is performed at the level of single organisations. On such a basis, we suggest that the microscopic analysis of shock resilience is a fruitful avenue for future research and, in particular, the tools and capabilities that organisations may exploit to be resilient. In parallel, a macroscopic analysis of everyday resilience may be interesting for identifying chronic and/or long-run stressors that may threaten the entire health sector and for defining common action plans and policies to appropriately respond to them. The few contributions in this field deal with the health challenges related to climate change and military conflict in developing countries; indeed, in both cases, health risks arise from large-scale stressors affecting the systems as a whole.

Third, in the context of everyday resilience, most of the contributions focus on the role of managerial capabilities in fostering resilience against chronic stressors by neglecting the role played by physical resources, that is, human, economic, digital resources, and infrastructure.

Beyond these differences, several common paths for future research emerge, since the field remains insufficiently explored. First, resilience has been scarcely investigated from a cross-organisational point of view—that is, as the coordinated response of multiple actors cooperating for health service provision (e.g. hospitals, general practitioners, local health authorities, private companies). The only works going beyond the level of single organisations merely consider groups of organisations within the same territorial context (e.g. community or regional health systems) to provide comparative analysis or aggregate statistics. Therein, each unit is independent of the others, and no interaction among them is considered. We suggest that, in future research, the lens could be moved towards *health ecosystems* to analyse how the cooperation mechanisms between single entities and their orchestration may strengthen resilience capabilities. In this regard, a focus on the role played by digital technologies would be of particular interest, as they are widely recognised as crucial resources that could facilitate the integration of the actors within health ecosystems and, hence, their dynamic capabilities.

Second, in both literature streams, the role played by single individuals or teams during a crisis is quite unexplored in the managerial-oriented body of research. The managerial-oriented literature recognises that in most organisations, as human capital is a fundamental part of their functioning, the characteristics of the individuals remarkably impact the resilience of the organisation [88]. Therefore, future research in the healthcare domain should also focus on strategies to increase their preparedness to operate in turbulent and unpredictable contexts. Hence, potential directions for future research in this area could include the identification of factors that influence individual and team resilience in the healthcare sector, the definition of organisational strategies that could be enacted to foster resilient teams and staff (e.g. education or training programmes), and the resources that could be used to build resilience. Beyond the role of single employees, leadership capabilities should also be better explored to identify the ability that a leader should develop to face challenges arising during acute disruptions or unpredictable events and to effectively drive organisations towards new equilibria. In this regard, the entrepreneurship literature stresses that the resilience of entrepreneurs and managers—that is, the leadership roles—is inseparable from the resilience of the organisation. Recently, the concept of resilient leadership has gained momentum but not in the healthcare area. How to become resilient leaders in terms of what to do at different times, given different priorities, and in the face of a crisis [89] could also be beneficial in healthcare settings where leadership roles are invariably present and have the responsibility of maintaining the equilibrium of their team members.

These interpersonal dynamics are linked to the third interesting area for future research related to the resources analysed to nurture resilience, particularly the soft resources that have received minor attention. It seems that, so far, the literature has not considered the social norms, the behavioural aspects, and the social interactions as crucial to foster resilience. Following the line of the literature on quality management [90], future research should shed light on these aspects to understand if and how they may increase resilience. For example, top management commitment and motivation or employee involvement and empowerment, which points to the behavioural aspect of management, and patient focus or satisfaction, which is more related to the external environment, could be worth examining.

Fourth, categorising the articles based on the country shows significant polarisation with reference to the type of crisis they deal with and the approach to resilience. Specifically, most studies on everyday resilience refer to applications and cases in developed countries, whereas those considering developing countries mainly deal with acute shocks, apart from recent studies about the COVID-19 pandemic. A twofold motivation may explain this circumstance. First, in recent years, developing countries have been affected by several acute events, such as infectious diseases (e.g. Ebola outbreak) and natural disasters, that stimulated studies on the topic. Second, there is a considerable gap between the economic conditions of the two groups of countries and the service levels provided by their health systems. This implies a different management culture and priority agendas, which reflect an almost exclusive reactive approach in developing countries. For example, the culture of risk management and patient safety is much more widespread in developed countries than in developing ones. This polarisation confirms a divide in the culture of resilience in the two considered contexts, which may be better explored in future research. Possible avenues in this area could consider strategies to exploit the lessons learned in developed countries to accelerate the diffusion of resilient culture and foster the adoption of practices to respond to everyday stressors.

**Fig. 5:**
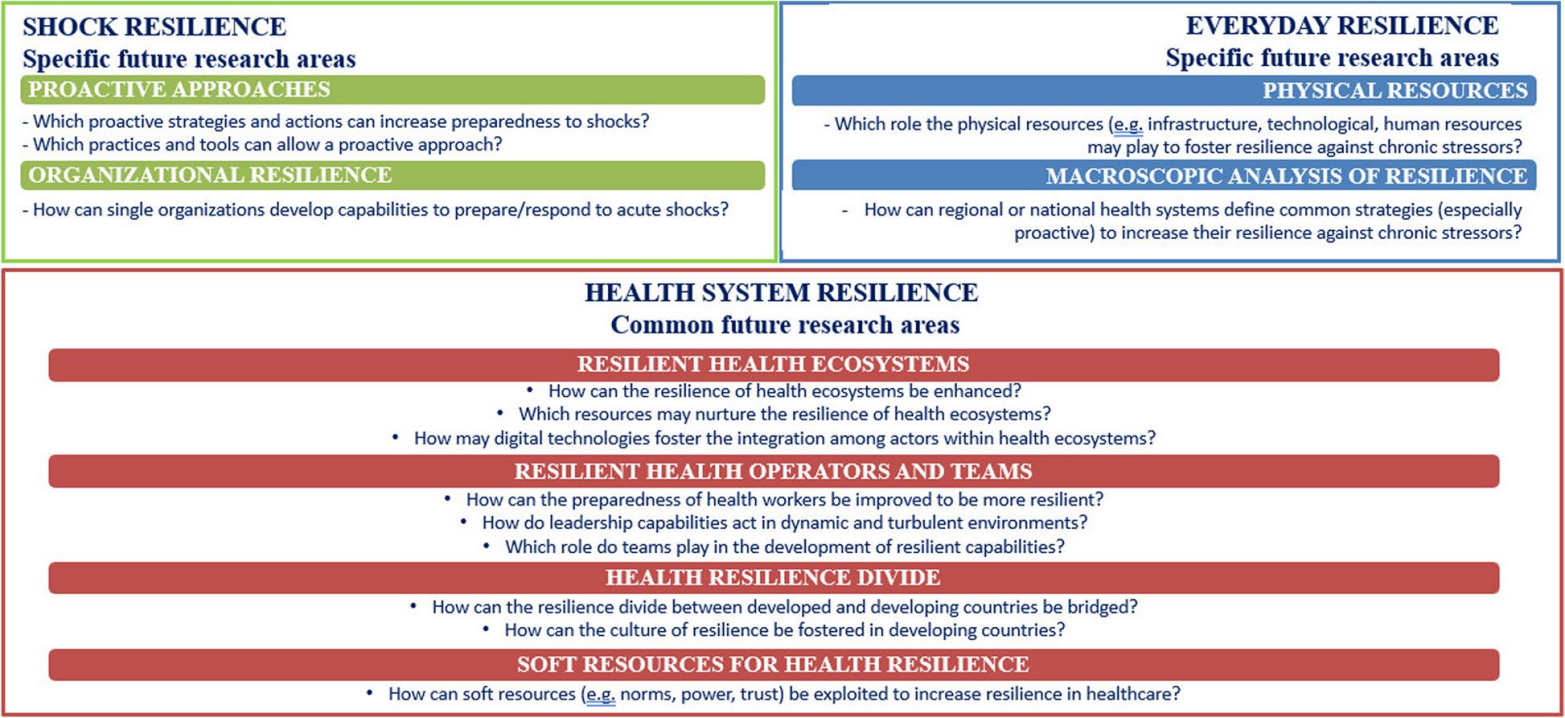
Possible research questions for future research

To summarise the research directions that emerge from this review, Fig. 5 illustrates a tentative research agenda proposing the main research questions within the several areas highlighted as potential “white spaces” for future research.

## Conclusion

This systematic review focuses on the management of resilience in healthcare and intends to provide a knowledge base for further scientific development in this important research area. In so doing, this research builds on previous theoretical research to design a classification framework to be used to systematise empirical research, thus offering an overview of existing evidence and proposing promising avenues for future research. In doing so, it offers both theoretical and practical implications. From a theoretical perspective, this review identifies the main dimensions to classify empirical research in the domain of resilience in healthcare, namely the typology of crisis to deal with and the level of analysis as the two main ones, and the country context, the resilience approach, and the resources to nurture resilience as the three complementary ones. The resulting classification framework is based on theoretical articles in this field; therefore, it constitutes a sound and well-grounded reference framework that not only allows for organising existing literature, as recently called for [13] but also provides a reference framework for future studies to position themselves consistently, thus allowing an organised development of the literature. Moreover, by scrutinising articles along the introduced dimensions, several gaps emerge, which opens interesting paths for future research. Accordingly, some research questions have been put forward for the shock resilience and everyday resilience streams, as well as for considering the whole body of knowledge.

This study also provides relevant practical contributions, offering a knowledge base supporting decision making in healthcare. A solid knowledge and understanding of the principles underlying resilient management can facilitate leaders of health organisations in their decision making in the face of uncertain events. By identifying the empirical approaches used, this study highlights different approaches and resources for increasing resilience at different levels of the system. For example, managers with greater awareness of this issue can be guided in designing resilience actions for both shocks (i.e. shock resilience) and chronic stressors (i.e. everyday resilience) from the micro to the macro level of the system. Sometimes, they repeat old procedures/protocols to address new problems, even when these could be solved only by using different approaches and/or involving different resources that are sometimes unknown. In addition, these findings could provide insights into designing new training pathways for all health professionals with respect to the specific contingencies of the systems in which they operate. Furthermore, factors that proved efficient in responding reactively to acute shocks at the organisational level could represent specific indications about how to proactively engage to be ready to face new acute shocks. For example, the issue of technological tools and telemedicine could be an area of potentially fruitful investment by decision making operating in healthcare organisations, besides other management tools already tested to react to chronic stressors. Further, an action on employees could be useful, with particular reference to specific training to upgrade their capabilities and possibly align them with the evolution of the tools and systems used in the organisation. This could be a way to involve all levels of healthcare organisations in a process of change that is difficult to accept in a sudden, but rather needs participation, commitment, and a sense of belonging and appreciation that managers should be able to convey to their personnel.

Overall, the study demonstrated the importance of developing specific knowledge to foster a proper conceptualisation of resilience in healthcare. We also recognise that this research has some limitations: (i) we used only WoS to retrieve articles; therefore, the query could be enlarged to other databases; and (ii) we limited our study to the management domain, which is a peculiarity of such studies as ours; however, our framework could be extended to other fields.

## Supplementary Information

**Additional file 1: Appendix **[91–145].

## Data Availability

In our case, data are the articles belonging to the final sample analysed for the literature review. If required, it is available. Please, contact the Corresponding author to request the data from this study.

## Footnotes

1 High-income countries following *The World Bank* classification (2021) available at: https://datahelpdesk.worldbank.org /knowledgebase/articles/906519.
