# Supplementary material for: A management perspective on resilience in healthcare: a framework and avenues for future research
Source: BMC Health Serv Res. 2023 Jul 19;23:774. doi: 10.1186/s12913-023-09701-3 (PMC10357696; doi:10.1186/s12913-023-09701-3)
Supplement: Supplementary file 1 — Additional file 1: Appendix [91–145]. [file 12913_2023_9701_MOESM1_ESM.docx]

**Appendix A**

|  |  |  | **LEVEL OF ANALYSIS** | | | | |
| --- | --- | --- | --- | --- | --- | --- | --- |
|  |  |  | **M1**  **(INDIVIDUAL)** | **M2**  **(TEAM)** | **M3**  **(ORGANIZATION)** | **M4**  **(NETWORK)** | **M5**  **(COUNTRY)** |
| **SHOCK** | **REACTIVE APPROACH** | **HARDWARE RESOURCES** |  |  | [50], [53], [91], [92]*, [93], [94], [95], [96], [97] | [45], [99] | [46] |
|  |  | **ORGANIZATION AND MANAGEMENT** | **Management Tool** | **Management Tool** | **Management Tool**  [52], [98]*, [100]* | **Management Tool**  [36], [101]* | **Management Tool**  [27]*, [44], [48]* |
|  |  |  | **Capabilities**  [54], [56], [102]* | **Capabilities**  [54] | **Capabilities**  [49]*, [54], [98]*, [103]* | **Capabilities**  [37]*, [47] | **Capabilities**  [42], [43] |
|  |  |  | **Governance**  [56] | **Governance** | **Governance**  [53], [93], [96], [97] | **Governance**  [34]*, [38]*, [40]*, [41], [104], [105], [106] | **Governance**  [42], [43], [107]*, [108]*, [109], [110]*, [111] |
|  |  | **SOCIAL FACTORS** | [55]*, [63], [112]*, [113]*, [23]*, [114]* |  | [98]* | [8] [39]* |  |

**TABLE A1 –Map of contributions analyzing REACTIVE APPROACHES to COPE with ACUTE SHOCKS on the basis of the considered level of analysis, resources and country context**

**[Note that the symbol * indicates that the article deal with developing countries]**

|  |  |  | **LEVEL OF ANALYSIS** | | | | |
| --- | --- | --- | --- | --- | --- | --- | --- |
|  |  |  | **M1**  **(INDIVIDUAL)** | **M2**  **(TEAM)** | **M3**  **(ORGANIZATION)** | **M4**  **(NETWORK)** | **M5**  **(COUNTRY)** |
| **SHOCK** | **PROACTIVE APPROACH** | **HARDWARE RESOURCES** | [62] |  |  |  | [115]* |
|  |  | **ORGANIZATION AND MANAGEMENT** | **Management Tool** | **Management Tool** | **Management Tool**  [64] | **Management Tool**  [65], [116]* | **Management Tool** |
|  |  |  | **Capabilities** | **Capabilities** | **Capabilities** | **Capabilities** | **Capabilities**  [115]* |
|  |  |  | **Governance** | **Governance** | **Governance**  [117]* | **Governance**  [59], [29] | **Governance**  [61], [66]*, [118]* |
|  |  | **SOCIAL FACTORS** | [62] |  |  |  |  |

**TABLE A2 –Map of contributions analyzing PROACTIVE APPROACHES to COPE with ACUTE SHOCKS on the basis of the considered level of analysis, resources and country context**

**[Note that the symbol * indicates that the article deal with developing countries]**

|  |  |  | **LEVEL OF ANALYSIS** | | | | |
| --- | --- | --- | --- | --- | --- | --- | --- |
|  |  |  | **M1**  **(INDIVIDUAL)** | **M2**  **(TEAM)** | **M3**  **(ORGANIZATION)** | **M4**  **(NETWORK)** | **M5**  **(COUNTRY)** |
| **STRESSORS** | **REACTIVE APPROACH** | **HARDWARE RESOURCES** |  |  | [67], [68] |  |  |
|  |  | **ORGANIZATION AND MANAGEMENT** | **Management Tool** | **Management Tool** | **Management Tool** | **Management Tool** | **Management Tool**  [119]* |
|  |  |  | **Capabilities**  [70] | **Capabilities** | **Capabilities** | **Capabilities** | **Capabilities**  [120]* |
|  |  |  | **Governance** | **Governance** | **Governance** | **Governance** | **Governance**  [69], [66]*, [120]*, [122] |
|  |  | **SOCIAL FACTORS** |  |  |  | [121] |  |

**TABLE A3 –Map of contributions analyzing REACTIVE APPROACHES to COPE with CHRONIC STRESSORS on the basis of the considered level of analysis, resources and country context**

**[Note that the symbol * indicates that the article deal with developing countries]**

|  |  |  | **LEVEL OF ANALYSIS** | | | | |
| --- | --- | --- | --- | --- | --- | --- | --- |
|  |  |  | **M1**  **(INDIVIDUAL)** | **M2**  **(TEAM)** | **M3**  **(ORGANIZATION)** | **M4**  **(NETWORK)** | **M5**  **(COUNTRY)** |
| **STRESSORS** | **PROACTIVE APPROACH** | **HARDWARE RESOURCES** |  |  | [51] , [127], [128] | [136] | [82]* |
|  |  | **ORGANIZATION AND MANAGEMENT** | **Management Tool** | **Management Tool** | **Management Tool**  [71], [72], [73], [74], [76], [77], [78], [129], [130] | **Management Tool**  [81]*, [137], [138] | **Management Tool**  [141] |
|  |  |  | **Capabilities** | **Capabilities** | **Capabilities** [79], [80], [106], [127]*, [131]*, [132]*, [133]* | **Capabilities** | **Capabilities**  [82]* |
|  |  |  | **Governance** | **Governance** | **Governance** | **Governance**  [83]*, [139], [140] | **Governance**  [140], [142], [143], [144], [145] |
|  |  | **SOCIAL FACTORS** | [123], [124] | [125], [126] | [134], [135] |  |  |

**TABLE A4 –Map of contributions analyzing PROACTIVE APPROACHES to COPE with CHRONIC STRESSORS on the basis of the considered level of analysis, resources and country context**

**[Note that the symbol * indicates that the article deal with developing countries]**
